# Inherent Anharmonicity of Harmonic Solids

**DOI:** 10.34133/2022/9786705

**Published:** 2022-04-29

**Authors:** Matthias T. Agne, Shashwat Anand, G. Jeffrey Snyder

**Affiliations:** Department Materials Science and Engineering, Northwestern University, Evanston, IL, USA

## Abstract

Atomic vibrations, in the form of phonons, are foundational in describing the thermal behavior of materials. The possible frequencies of phonons in materials are governed by the complex bonding between atoms, which is physically represented by a spring-mass model that can account for interactions (spring forces) between the atoms (masses). The lowest-order, *harmonic*, approximation only considers linear forces between atoms and is thought incapable of explaining phenomena like thermal expansion and thermal conductivity, which are attributed to nonlinear, *anharmonic*, interactions. Here, we show that the kinetic energy of atoms in a solid produces a pressure much like the kinetic energy of atoms in a gas does. This vibrational or *phonon pressure* naturally increases with temperature, as it does in a gas and therefore results in a thermal expansion. Because thermal expansion thermodynamically defines a Grüneisen parameter γ, which is a typical metric of anharmonicity, we show that even a harmonic solid will necessarily have some anharmonicity. A consequence of this phonon pressure model is a harmonic estimation of the Grüneisen parameter as $\gamma \approx \left(3/2\right)\left(3-4x^{2}\right)/\left(1+2x^{2}\right)$, where $x=v_{\text{t}}/v_{\text{l}}$ is the ratio of the transverse and longitudinal speeds of sound. We demonstrate the immediate utility of this model by developing a high-throughput harmonic estimate of lattice thermal conductivity that is comparable to other state-of-the-art estimations. By linking harmonic and anharmonic properties explicitly, this study provokes new ideas about the fundamental nature of anharmonicity, while also providing a basis for new material engineering design metrics.

## 1. Introduction

The characterization and understanding of atomic bonding has been the subject of solid state physics and physical chemistry for more than a century, and even still there are new notions about the nature of chemical bonds in solids [1]. Fundamental to the description of a bond is how it deforms when a force is applied. Although most materials exhibit Hookean elastic behavior (i.e., a linear stress-strain relation) for small atomic displacements, this often breaks down at larger displacements. And yet, this nonlinear or anharmonic behavior is essential to the material properties relevant to modern energy technologies, such as thermal conductivity [2], atomic/ionic diffusion [3], thermal stability [4], defect formation [5], ferroelectricity [6], and superconductivity [7]. For thermoelectric materials in particular, anharmonicity is seen as crucial for low thermal conductivity and high thermoelectric performance [2, 8–10].

The spring-mass model of atomic bonding has been an indispensable tool, providing a clear conceptual link between classical and quantum mechanics [11]. In this model, the complicated interactions between atoms (masses) are attributed to spring-like forces. Specifically, the key aspects of bonding are described by an n-degree Taylor expansion of the interatomic potential about the equilibrium position of the atoms. Then, the total potential energy of the material can be written in terms of the displacement u of atoms α, β, etc. from their equilibrium positions in Cartesian coordinates (indexed by i,j,k) as [12]

(1)U=U0+∑α∑i−fiαuiα+12!∑α,β∑i,jCijαβuiαujβ+13!∑α,β,γ∑i,j,kCijkαβγuiαujβukγ+⋯.

Here, $U_{0}$ accounts for any initial potential energy already contained by the bonds (springs) at equilibrium and the linear term ($-f_{i}^{\alpha }={\left(\partial U/\partial u_{i}^{\alpha }\right)}_{u_{i}^{\alpha }=0}$) is zero since there is no net force on any atom at equilibrium (by definition). The “harmonic” term (with force constants $C_{ij}^{\alpha \beta }$) is the lowest-order term that describes changes in the potential energy when an atom is displaced from its equilibrium position and can be used to obtain the fundamental frequencies of vibration (i.e., phonon eigenmodes). Cubic (i.e., $C_{ijk}^{\alpha \beta \gamma }$) and higher-order “anharmonic” terms account for nonlinear forces between atoms. Implicitly, each term ($U_{0}$, f, C, and u) in equation (1) is temperature dependent since the force constants and equilibrium positions of the atoms can change with temperature.

The generality of equation (1) has made it the starting point for theoretical and computational assessments of diverse material behaviors [11–13]. But it becomes computationally expensive to consider both higher-order terms in atomic displacement and the temperature dependence of the potential. The thermodynamic Maxwell relation, however, between entropy S, pressure P, volume V, and temperature T,

(2)∂S∂VT=∂P∂TV,

subsequently shows that isothermal material properties can be related to temperature-dependent properties (the subscripts indicate which variable is held constant). It is not surprising then that a description of thermal expansion (i.e., the quasi-harmonic model [14]) can be obtained using the Taylor expansion (equation (1)) at 0 K, since ${\left(\partial P/\partial T\right)}_{V}$ is directly related to the bulk modulus $B=-{\left(\partial P/\partial \ln V\right)}_{\text{T}}$ and thermal expansion coefficient $\alpha ={\left(\partial \ln V/\partial T\right)}_{P}$ as

(3)∂P∂TV=−∂P∂lnVT∂lnV∂TP=Bα.

**Figure 1:**
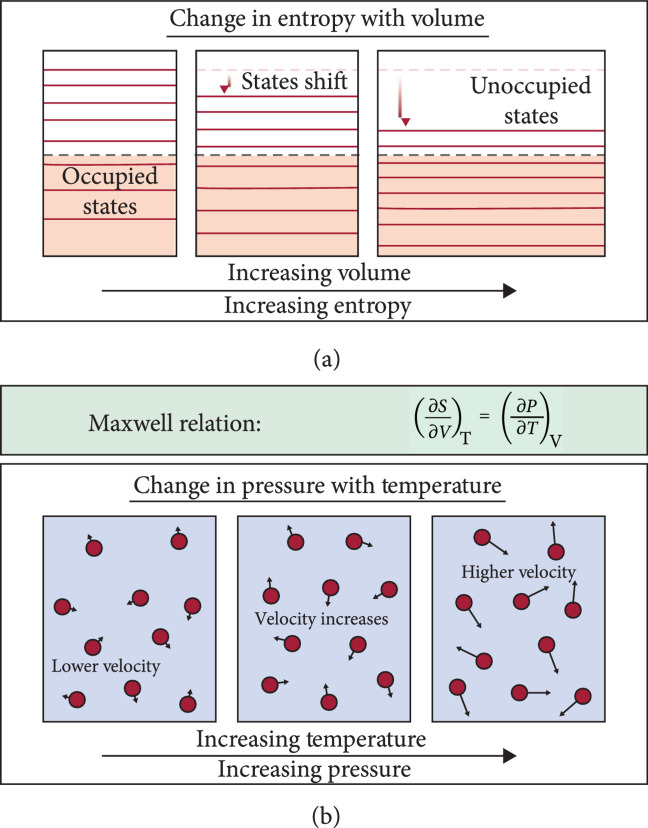
Two different paths to understanding thermal expansion. While thermodynamically equivalent (equation (2)), the current explanation of thermal expansion is through the perspective that vibrational modes shift as the volume changes (a). In this picture, anharmonicity is required for vibrational states to shift. Alternatively, the change in pressure due to changes in temperature can be considered (b), as is often done for gases. Pressure is related to the kinetic energy (velocity) of the atoms, and anharmonicity is not required in the lowest-order description of atomic movement in solids.

In the quasi-harmonic approximation, the change in vibrational entropy with volume is attributed to changes in phonon frequencies $\omega_{i}$ with volume, i.e., ${\left(\partial S/\partial V\right)}_{T}=\sum_{i}{\left(\partial S/\partial \omega_{i}\right)}_{T}{\left(\partial \omega_{i}/\partial V\right)}_{T}$. From this perspective (e.g., Figure 1), anharmonic terms are necessary in equation (1) for ${\left(\partial \omega_{i}/\partial V\right)}_{T}\neq 0$ [15], leading to the conclusion that harmonic solids cannot have thermal expansion.

Utilizing the phonon entropy (e.g., the quasi-harmonic approximation) however is but one approach to modeling the physics of anharmonicity (Figure 1). Alternatively, a mechanistic description of the pressure in solids could be used. This is easily done for an ideal gas but has not been considered for solids. The pressure exerted by an ideal gas is due to its kinetic energy. Atoms in a solid have the same form of kinetic energy as an ideal gas and should therefore have a similar pressure. Importantly, the kinetic energy of atoms inside a solid will change with temperature with or without any change in the vibrational frequencies. This is to say that a mechanical model of pressure in solids would not necessarily require anharmonic terms to be included in the potential.

Herein, we provide an intuitive (mechanical) understanding of thermal expansion that also suggests an inherent connection between harmonic and anharmonic aspects of bonding in real solids. The specific nature of thermal expansion, thermal conductivity, and other “anharmonic” material properties is at the forefront of solid-state materials research [16], as are methods to predict/calculate these properties *en masse* [17–19]. Consequently, a mechanistic understanding of vibrational pressure in solids has broad implications for engineering design metrics of diverse material phenomena, such as negative thermal expansion, ultralow thermal conductivity, and superconductivity. We demonstrate this utility by developing a harmonic estimate of the lattice thermal conductivity that is suitable for high-throughput screening of materials.

## 2. Results and Discussion

**Figure 2:**
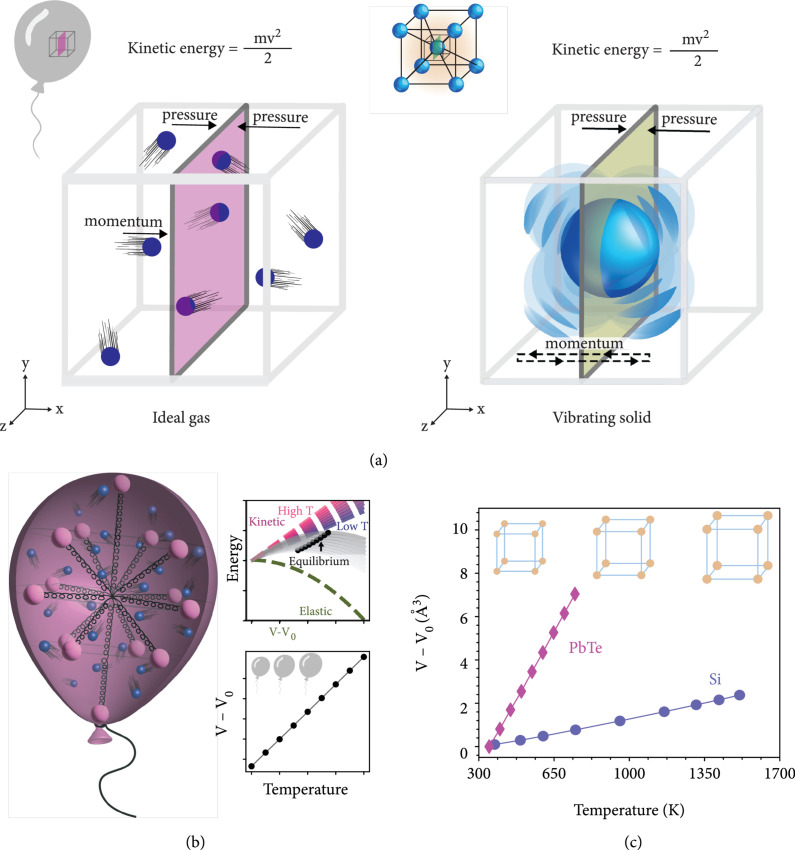
Depiction of pressure due to kinetic energy and its effect on equilibrium volume with temperature. (a) The pressure due to kinetic energy can be derived by considering the momentum flux through a fixed imaginary surface (e.g., pink and green planes in figure). Since the (time averaged) flux of momentum is equal in both directions, the (thermodynamic) pressure exerted on both sides of the surface is also equal. From this perspective, vibrating atoms in a solid exert a pressure on the rest of the solid just as gas particles exert a pressure on their container. (b) An elastic balloon-like container filled with an ideal gas, corresponding to the case described by equation (4). Here, radially distributed springs define the elasticity of the container. The temperature dependence of the equilibrium volume is due to the balance between the temperature-dependent kinetic energy of the gas (blue balls) and the corresponding elastic response of the material. As an analogy, the elastic balloon-like container represents the bonding between atoms holding the material together and the ideal gas represents the atomic vibrations pushing the solid outward. (c) The equilibrium volume of solids [22, 23] increases linearly with increasing temperature (at high temperature) and can be attributed to an analogous mechanical mechanism as the thermal expansion of the ideal gas in an elastic medium.

It is well known that a monatomic ideal gas at volume V exerts a pressure P (force/area) that is proportional to its average internal energy, PV=2E/3. When combined with the equipartition theorem ($E\sim k_{\text{B}}T$), this relation gives the microscopic origin of the ideal gas law, PV=NRT. In general, pressures arise as a result of the confinement of kinetic energy. Consequently, diverse physical systems from ideal gases to laser beams [20] and ultrasound acoustic waves [21] all produce pressures that are proportional to their kinetic energy density. A connection with the Umov-Poynting vector should also be noted. Each of these pressures can be determined by considering the flux of linear momentum (i.e., kinetic energy) through an arbitrary plane representing a confining surface (Figure 2(a) and Supplemental Note 1). In the realistic case where the kinetic particles are actually confined by an elastic medium, there is an associated pressure (stress) provided by the medium as it deforms (i.e., strain≠0) in response to interacting with the particles.

For the same reasons, the kinetic energy of atomic vibrations gives rise to pressures in solids that can be thought of as phonon pressure. In fact, analogous to the ideal gas pressure, this phonon pressure is temperature dependent due to the change in kinetic energy with temperature. This is to say that, even for a harmonic solid, there is a pressure that can mechanistically facilitate thermal expansion (see Methods). For the sum of forces inside the solid to be zero at equilibrium (e.g., the linear term $f_{i}^{\alpha }$ in equation (1)), the divergence of the stress tensor must be zero. Thus, the force exerted by the atomic vibrations must be compensated by an elastic restoring force that is proportional to the change in the equilibrium position. This is essentially the same physics that describes mechanical equilibrium in an elastic balloon-like container filled with an ideal gas.

To illustrate, consider a gas kept at a finite pressure in equilibrium with an elastic container (Figure 2(b)). An important distinction here is that the elasticity of the container is defined by radially distributed springs originating at the center of the container and not by the surface of the container. The elastic response of the container is characterized by the bulk modulus B so that the total pressure of the system at volume V can be related as

(4)Ptotal=Pkinetic+Pelastic=NRTV−BV−V0V=0.

The volume that satisfies this equilibrium condition is $\left(V-V_{0}\right)=\text{NRT}/B$, which increases linearly with temperature. This is known to be the case according to Charles’ law (i.e., at constant pressure) and also holds for the case of elastic pressure considered here. Experimentally, it is well demonstrated that the volume of solids is linearly proportional to temperature (Figure 2(c)) when the kinetic energy of atomic vibrations increases linearly with temperature (i.e., at high temperature). At lower temperatures, the kinetic energy of atomic vibrations is not linear with temperature, as described by Bose-Einstein statistics, but the form of equation (4) is retained (see equation (9)).

The expansion of a balloon-like container with temperature is the natural result of balancing the temperature-dependent pressure of the kinetic gas with the elastic pressure in the container (equation (4) and Figure 2(b)). This condition of static equilibrium (i.e., $P_{\text{total}}=0$) is determined by minimizing the internal energy of a system with an interface (comprised of the temperature-dependent kinetic energy plus total elastic energy) at a given temperature (Figure 2(b) and Supplemental Note 2). Because vibrating atoms in a solid have the same form of momentum flux (kinetic energy) as a gas, they must also produce an outward pressure to expand. Unlike a gas, which needs external confinement (e.g., the balloon), the balancing force creating the mechanical equilibrium (net force = 0) in a solid must be from the interatomic forces (represented by springs).

This is to say that thermal expansion can be described within the harmonic approximation. Specifically, the present theory uses the phonons found from the harmonic approximation to calculate the pressure exerted by atoms on the rest of the solid. There is a corresponding volume (strain) that satisfies the equilibrium condition at each temperature (analogous to equation (4)). The thermal expansion coefficient is found from summing the individual contributions of all the vibrational modes (see Methods).

**Figure 3:**
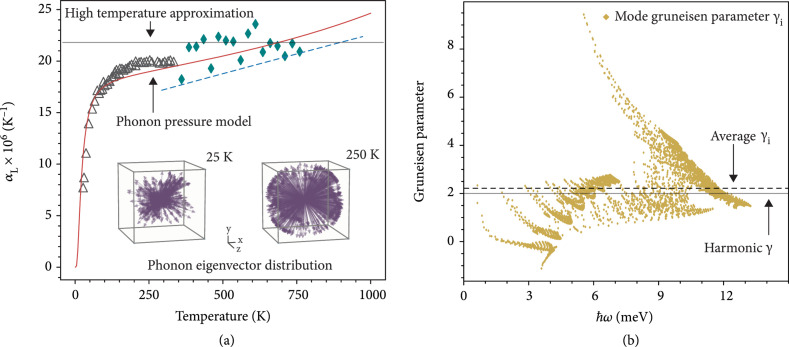
Thermal expansion coefficient and Grüneisen parameters of lead telluride, PbTe. (a) Coefficient of linear thermal expansion $\alpha_{\text{L}}$ for PbTe estimated using the “phonon pressure model” of thermal expansion (see Methods) calculated from harmonic eigenmodes and eigenvectors found from density functional theory (solid orange line) compared with experimental results (triangles [24], diamonds [22], and dashed line [25]). The “high-temperature approximation” is found from a simplified analytical model (see Methods) and is defined as $\alpha_{\text{L}}=3k_{\text{B}}/2\bar{m}v_{\text{s}}^{2}$, where $k_{\text{B}}$ is Boltzmann’s constant, $\bar{m}=2.8\times 10^{-25}$ kg atom^-1^ is the average atomic mass, and $v_{\text{s}}=1850$ m s^-1^ is the average speed of sound. The inset illustrates how phonon modes contribute to the vibrational pressure exerted by an atom differently at different temperatures due to the number of phonons that are excited. Here, the real space direction of the atom vibrations (i.e., phonon eigenvectors) is shown by arrows. The length of the arrows is scaled by the heat capacity of the phonon mode. (b) Mode Grüneisen parameters $\gamma_{i}$ of PbTe calculated from density functional theory (gold diamonds) and their average value, compared with the estimated “harmonic Grüneisen parameter” (see Methods).

Remarkably, this intuitive picture of thermal expansion largely captures both the magnitude and temperature dependence of experimental observations (Figure 3(a)). It should be reiterated that this estimation of the thermal expansion coefficient is determined solely using harmonic vibrational states in conjunction with the mechanical concept of phonon pressure (e.g., see inset of Figure 3(a)). The relevance of lead telluride, PbTe, as a high-efficiency thermoelectric material makes this example particularly interesting, since understanding its vibrational properties is fundamental to engineering its thermal conductivity [26]. As PbTe is considered an archetype of anharmonic materials [8] and is known to have complex (higher-order) bonding interactions [27], it is unexpected that a harmonic model could capture an anharmonic property like the thermal expansion behavior so well. In the quasi-harmonic approximation, the large thermal expansion coefficient is attributed to the relatively large positive mode Grüneisen parameters ($\gamma_{i}=-{\left(\partial \ln\omega_{i}/\partial \ln V\right)}_{\text{T}}$) at all frequencies (Figure 3(b)). Here, the phonon pressure makes the solid expand, corresponding to $\gamma_{i}>0$. The close agreement between the two perspectives (compare “harmonic γ” and “average $\gamma_{i}$” in Figure 3(b)) is indicative of an underlying connection (e.g., Figure 1). In the present perspective, the large thermal expansion coefficient in PbTe is thus explained by the large ratio of phonon pressure to the elastic restoring force.

It should be noted that, in the present calculations, atoms were considered to vibrate independent of each other. However, the coordinated movements of neighboring atoms are likely important in some cases, particularly in negative or anomalous thermal expansion materials [28, 29]. Nevertheless, internal pressure is suggested to play an important role in the dynamics of negative thermal expansion in ScF_3_ [30]. The influence of higher-order anharmonicity may also contribute to the behavior of thermal expansion [14]. Thus, further considerations of atomic structure and bonding could lead to a better understanding of the relation between the present phonon pressure perspective and the mode Grüneisen parameters found from quantum mechanics, which may provide insights into the origin of negative thermal expansion in some solids.

**Figure 4:**
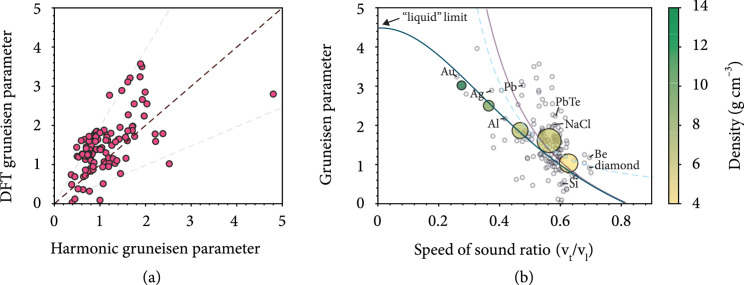
Apparent relations between “anharmonic” and harmonic properties of solids. (a) An equal plot comparing the “DFT Grüneisen parameter” (i.e., the average mode Grüneisen parameter) with the “harmonic Grüneisen parameter” (i.e., the thermodynamic Grüneisen parameter estimated from the harmonic model of thermal expansion) for 119 compounds. Light grey dashed lines indicate a factor of 2 from the equal line. (b) The thermodynamic Grüneisen parameter estimated from equation (7) in terms of the speed of sound ratio $x=v_{\text{t}}/v_{\text{l}}$ (using the RMS speed of sound, which is equation (8), gives the dark blue line, and using the bulk modulus and $v_{\text{s}}$ described by Anderson [34] gives the purple line, which diverges as x⟶0 and can be found in Supplemental Note 3) compared with the previous theory given by Druyvesteyn [35] (dashed blue line) and the DFT calculated thermodynamic Grüneisen parameters (average $\gamma_{i}$) of individual materials (light grey circles) as well as the average Grüneisen parameters (yellow-green circles) of materials binned according to their speed of sound ratio (bins: [0.2,0.3), [0.3,0.4), [0.4,0.5), [0.5,0.6), and [0.6,0.7]). The area of the marker is related to the number of materials it represents, and the color represents the average density of those materials (Supplemental Note 4). The “liquid” limit for this model of solids is the case where the transverse speed of sound goes to zero (Poisson's ratio = 0.5). The other thermodynamic limit ($v_{\text{t}}/v_{\text{l}}=\sqrt{3}/2$, Poisson's ration = -1) is the point where equation (8) gives γ=0.

Complex bonding interactions in real solids necessarily include anharmonic terms (i.e., nonlinear forces). Here, we show that the lowest-order harmonic description is related to those higher-order effects (Figures 3 and 4) but does not preclude additional anharmonic considerations that may be important. Specifically, the harmonic theory of thermal expansion is thermodynamically connected to anharmonicity through the Grüneisen relation. The thermodynamic Grüneisen parameter γ is a restatement of equation (2) that can be written as [15]

(5)γ=αBcV,

where the (volumetric) heat capacity $c_{\text{V}}$ approaches the Dulong-Petit value (3 $k_{\text{B}}$/atom) at high temperature. Then, the “harmonic Grüneisen parameter” can be calculated in the high temperature limit using the phonon pressure model of thermal expansion in conjunction with the bulk modulus determined by density functional theory (see Methods). This scalar “harmonic Grüneisen parameter” is compared with the scalar “DFT Grüneisen parameter” found by averaging the mode Grüneisen parameters $\gamma_{i}$ (Figure 4(a)). The overall agreement (within a factor of ~2) corresponds to the agreement between their respective thermal expansion estimations. The important point, however, is that the vibrational properties of harmonic phonon modes can be related to their own anharmonicity at the thermodynamic level.

Using the theory of phonon pressure, it is also possible to show that the thermodynamic Grüneisen parameter (average $\gamma_{i}$) can be estimated from the ratio of the bulk-averaged (i.e., isotropic polycrystalline equivalent) transverse and longitudinal speeds of sound ($x=v_{\text{t}}/v_{\text{l}}$, see equations (16) and (17)) (Figure 4(b)) or equivalently the isotropic Poisson’s ratio. The connection is plausible given that Poisson’s ratio is a fundamental metric of material behavior when strained elastically [31]. To arrive at this analytic result, we use a simplified form of the vibrational pressure model (see Methods) to obtain the thermal expansion coefficient α in terms of the heat capacity $c_{\text{V}}$, density ρ, and speed of sound $v_{\text{s}}$ as

(6)α≈32cVρvs2.

Substituting this result into the thermodynamic definition of the Grüneisen parameter (equation (5)) yields

(7)γ≈32Bρvs2,

which, when $v_{\text{s}}$ is considered to be the root mean square speed of sound (i.e., $v_{\text{s}}^{2}=\left(2v_{\text{t}}^{2}+v_{\text{l}}^{2}\right)/3$), can be written as (Supplemental Note 3)

(8)γ≈323−4x21+2x2,

where $x=v_{\text{t}}/v_{\text{l}}$. This form of the Grüneisen parameter (equations (7) and (8)) has been obtained previously [32, 33] but was based on a different assumption about the nature of vibrational pressure. In that work, the temperature dependence of vibrational pressure was not considered [32].

It is easily acknowledged that this approximation for the Grüneisen parameter (equation (8), dark blue line in Figure 4(b)) is quite crude given the scatter in the data (grey circles in Figure 4(b)). But its usefulness is more apparent when comparing with the average Grüneisen parameter of materials with similar speed of sound ratios (yellow-green circles in Figure 4(b)), demonstrating that there is an intimate connection between material chemistry, elasticity, and anharmonicity that has not been fully explored. For one, equation (8) indicates that solids that are more “liquid-like” (i.e., x⟶0) are expected to be more anharmonic. This corresponds to the large Grüneisen parameter in metals like gold, silver, and lead, where the atomic structure and bonding are often described as positively charged ions held together by a sea of negatively charged electrons—the reason being that the electrons are delocalized and can be easily rearranged like a liquid. Conversely, the Grüneisen parameter is much lower in highly directional, covalently bonded materials like diamond and silicon, when x is closer to the upper thermodynamic limit of $\sqrt{3}/2$. In this respect, beryllium, which has unusual elastic properties for a metallic element [36], also has a Grüneisen parameter that is in good agreement with the model. Another relation between γ and Poisson’s ratio [35], derived from analytical considerations of an anharmonic interatomic potential, indicates that γ diverges as x⟶0 but gives similar estimates as equation (8) for most materials (0.4<x<0.7, dashed blue line in Figure 4(b)). Using a different average, $v_{\text{s}}$ (i.e., a harmonic mean [34]) in equation (7) will also cause γ to diverge since $v_{\text{s}}⟶0$ as $v_{\text{t}}⟶0$ (purple line in Figure 4(b), Supplemental Note 3), suggesting that the root mean square speed of sound better captures the nature of the elastic pressure.

Altogether, this relation (equation (8)) provides a simple physical justification for the chemist’s intuition that the anharmonicity of solids is related to the type of bond and atomic coordination (density) [9, 37, 38], nor would it be surprising if more intricate connections were found between elasticity, atomic structure, and anharmonicity through modern machine learning methods.

**Figure 5:**
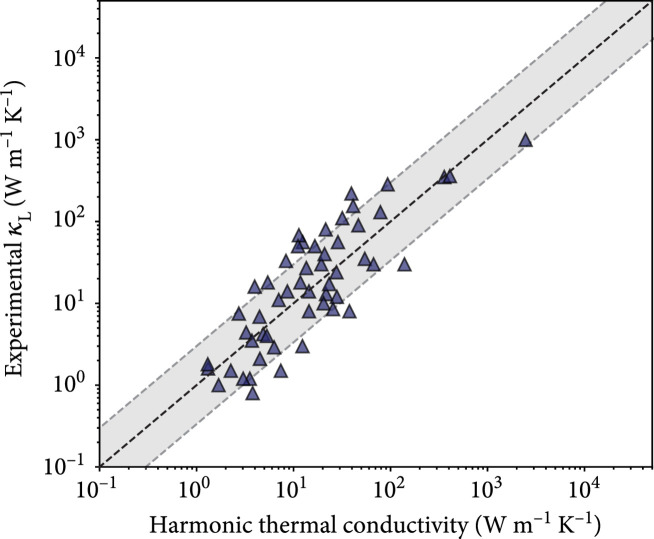
Harmonic estimation of lattice thermal conductivity. Here, the experimental values of lattice thermal conductivity $\kappa_{\text{L}}$ at 300 K for 53 previously reported compounds [37] were estimated using only harmonic descriptors in a semiempirical model (equation (15) and Supplemental Note 5). The grey shaded region spans a factor of 3 from the equal line and represents the approximate accuracy of the semiempirical model reported by Miller et al. [37].

This profound connection of the thermodynamic Grüneisen parameter with harmonic properties of solids means that transport phenomena like thermal conductivity and atomic diffusion may also be explained in terms of a harmonic potential. The temperature dependence of vibrational pressure corresponds to a temperature-dependent potential energy landscape such that interactions between vibrational modes (e.g., phonon-phonon scattering) may be accounted for within perturbation theory. However, further investigations are needed to reconcile the relations between harmonic and anharmonic terms in the interatomic potential. Nevertheless, having accessible estimates for thermal expansion (e.g., equation (6)) and the thermodynamic Grüneisen parameter allows for higher accuracy descriptions of the temperature-dependent molar volumes and constant pressure heat capacity [39] needed for the thermodynamic modeling that is crucial to the design of next generation materials [40]. For example, the harmonic Grüneisen parameter can be used in conjunction with a semiempirical model (see Methods) for high-throughput screening of lattice thermal conductivity (Figure 5 and Supplemental Note 5). This simple thermal conductivity model (equation (15)), which makes direct use of equation (8), shows the possibility of using a harmonic estimate of anharmonicity to accurately estimate materials properties. A main objective of this work is to introduce the harmonic Grüneisen parameter as a new materials descriptor of anharmonicity. Future analysis of materials properties like thermal conductivity, ionic conductivity, superconductivity, etc., may benefit greatly from a standardized and accessible metric of anharmonicity, such as equation (8). Thus, the consideration of phonon pressure in solids is not an esoteric exercise but provides a new basis for engineering design metrics that do not require the computationally expensive anharmonic calculations currently employed.

The concept of phonon pressure provides a physical mechanism for “anharmonic” behavior (i.e., thermal expansion) that thermodynamically relates harmonic and anharmonic aspects of bonding in solids. That is, harmonic solids have an inherent anharmonicity. In this perspective, the harmonic potential shifts with temperature to be centered around the equilibrium position determined by the force balance between vibrational pressure and the elasticity of the material (analogous to equation (4)). The success of the Temperature-Dependent Effective Potential (TDEP) method developed by Hellman and coworkers [41, 42] further supports the idea that there are aspects of anharmonicity that can be represented by a harmonic potential that changes with temperature. In the TDEP method, the equilibrium atomic position also changes with temperature through a force balance procedure. Instead of explicitly using the theory of vibrational pressure, the residual forces between those calculated from the model potential and those obtained by molecular dynamics simulation are minimized. The necessity of a temperature-dependent potential is apparent given that the Taylor expansion around equilibrium at 0 K is unable to reproduce finite temperature observations in some materials [41, 43].

## 3. Conclusion

Although the concept of energy flux as a pressure in elastic materials was first shown by Umov [44] and, somewhat later, both Brillouin [45] and Frenkel [46] proposed that thermal expansion in solids could be explained by vibrational pressure, the field of lattice dynamics largely left this mechanistic idea behind. As a result, it is commonly thought that harmonic solids cannot have thermal expansion and other properties assumed to be due entirely to anharmonic effects [47]. Here, the physical mechanism of phonon pressure leads to an explanation for thermal expansion based on only the lowest-order, harmonic approximation of solids. The connection of thermal expansion to the thermodynamic Grüneisen parameter, as well as other anharmonic behavior in solids, suggests that harmonic material descriptors may correlate with anharmonic properties more generally. Here, we show that the harmonic Grüneisen parameter, which is easily determined from experimental or computational methods, can be used as a metric of anharmonicity for engineering design and materials modeling. The insights gained from this fundamental shift in thought-paradigm may be used to better understand both basic thermodynamic properties of solids, including melting (e.g., the Lindemann melting criteria [48]), as well as the mechanisms of complex transport phenomena like superconductivity, ionic transport, and heat transport in defective materials.

## 4. Methods

### 4.1. Phonon Pressure Model

The divergence of the stress tensor must be zero at equilibrium. In many cases, this can be described by the balance of forces in a free body diagram. Here, we consider the sum of forces (normalized by a unit area) acting on atom α due to each phonon mode (having frequency ω, wave vector k, and branch index s). We consider mechanical equilibrium to occur when the normal component (in the $\hat{n}$-direction) of the vibrational (kinetic) stress $\sigma_{ks}^{\text{kinetic}}$ is exactly compensated by the normal component of the elastic stress $\sigma_{ks}^{\text{elastic}}$, such that the total normal stress $\sigma_{ks}^{\text{total}}$ (a scalar) due to the ks vibrational mode is

(9)σkstotal=σkskinetic+σkselastic=ħωksVafBEωks,Te∧ks·n∧2−ρvp2e^ks·n^εks=0,

where $\varepsilon_{ks}$ is the corresponding normal component of the strain. The strain is defined by the displacement of the atom from a 0 K reference position. The vibrational stress $\sigma_{ks}^{\text{kinetic}}$ due to the ks phonon mode (having the unit eigenvector ${\hat{e}}_{ks}$ that determines the motion of atom α) is proportional to the kinetic energy of each phonon ($\hbar \omega_{ks}/2$) as well as the number of phonons that are excited according to the Bose-Einstein distribution:

(10)fBEωks,T=expħωkskBT−1−1,

and $V_{\text{a}}$ is the atomic volume. Note the explicit temperature dependence of $f_{\text{BE}}$. Also note that the vibrational stress due to each phonon mode is found by considering its contribution to the normal component of the flux of linear momentum of atom α (Figure 2 and Supplemental Note 1).

The elastic stress $\sigma_{ks}^{\text{elastic}}$ acting against (compensating) the vibrational stress is defined by the elastic modulus ($\rho v_{\text{p}}^{2}$) governing the vibration, where ρ is the mass density of the solid and $v_{\text{p}}=\omega_{ks}/\left\|k\right\|$is the magnitude of the phase velocity of the phonon. The normal component of the strain $\varepsilon_{ks}$ (i.e., the strain in the $\hat{n}$-direction due to the ks vibrational mode) that results in mechanical equilibrium (equation (9)) is

(11)εks=ħωksVafBEωks,Te^ks·n^ρvp2.

The contribution of the ks vibrational mode to the normal component of the thermal expansion coefficient tensor is found using equation (9) and the thermodynamic relation

(12)∂εks∂Tσks=−∂σks/∂Tεks∂σks/∂εksT,

and the total thermal expansion coefficient in the $\hat{n}$-direction is found by summing over all vibrational modes:

(13)∂ε∂Tσ=∑k,s∂εks∂Tσks.

It should be noted that the rank 2 thermal expansion coefficient tensor can be constructed using appropriate values of $\hat{n}$. When $\hat{n}$ points only in the x-, y-, or z-direction, equation (13) corresponds to the linear thermal expansion coefficient $\alpha_{\text{L}}$ in that direction. The volumetric thermal expansion coefficient is the summation of the three linear thermal expansion coefficients, or in the case of materials with cubic symmetry like PbTe, $\alpha =3\alpha_{\text{L}}$.

Note that the above derivation is written explicitly for materials with one atom per primitive unit cell, but is easily generalized to many-atom unit cells by considering the partial pressures contributed by each atom.

The Grüneisen parameter can be calculated from the volumetric thermal expansion coefficient according to equation (5) and can be used to renormalize the frequencies $\omega_{ks}$ within the single Grüneisen parameter approximation (i.e., all vibrational modes have the same $\gamma_{i}$). This process can be done iteratively with temperature and, thus, phenomenologically accounts for the gentle increase in the thermal expansion coefficient at high temperatures (as shown in Figure 3(a)).

The “harmonic Grüneisen parameter” used in Figure 4 did not consider any renormalization of vibrational frequencies and was calculated using equations (9), (12), and (13) in the T⟶∞ limit.

A simplified analytic description of thermal expansion can also be derived from the concept of vibrational pressure (equation (6)). Here, an approximation for the frequency distribution of vibrational modes must be made (if the density of states is unknown), as well as the vibration direction. The Debye model of phonons as a dispersive continuum is often used to approximate the distribution of vibrational modes in solids and is applicable here. In the isotropic approximation the elastic properties of the solid are independent of direction and the atoms vibrate equally in all directions (i.e., the average incidence cosine $\left|{\hat{e}}_{ks}\cdot \hat{n}\right|$ is 1/2). Making use of these approximations, the linear thermal expansion coefficient $\alpha_{\text{L}}$ can be written as

(14)αL≈3kB4π2ρvs5∫0ωDω2ħωkBT2expħω/kBTexpħω/kBT−12dω,

where ρ is the density (kg m^-3^), $v_{\text{s}}$ is the average speed of sound (m s^-1^), and $\omega_{\text{D}}={\left(6\pi^{2}n\right)}^{1/3}v_{\text{s}}$ is the Debye frequency, which uses $v_{\text{s}}$ and the number density of atoms n (atoms m^-3^) to approximate the maximum frequency of vibration in the solid.

### 4.2. Density Functional Theory Calculations

Harmonic eigenmodes (phonons) and corresponding mode Grüneisen parameters $\gamma_{i}$ were found using density function theory methods. The compounds shown in Figure 4(a) were previously reported [18]. Additional calculations were undertaken to calculate thermodynamically averaged Grüneisen parameter for compounds with varied $v_{\text{t}}/v_{\text{l}}$ ratios as shown in Figure 4(b). The isotropically averaged speed of sounds ($v_{\text{t}}$ and $v_{\text{l}}$) were calculated using the christoffel code. [49] The elastic modulus tensor input for the code was calculated from the Density Functional Perturbation Theory (DFPT) [50, 51] capabilities implemented in the VASP code. [52] We used the PBEsol [53] formulation of the exchange–correlation energy functional derived under a generalized-gradient approximation (GGA). [54] Plane-wave basis sets were truncated at an energy cutoff of 500 eV, and a Γ- centered k-point mesh with a density of ~8000 k-points per reciprocal atom (KPPRA) was used. The electronic degrees of freedom in the self-consistent loop were converged to 10^-8>^ eV. All structures were relaxed with respect to cell vectors and their internal degrees of freedom until forces on all atoms were less than 0.1 eV nm^-1^. “DFT Grüneisen parameters” were found by thermodynamically averaging the mode Grüneisen parameters $\gamma_{i}$ in the high temperature limit such that they are weighted equally at all frequencies. The mode Grüneisen parameters were calculated using a finite difference method as implemented in phonopy. [12] For this, the phonon calculations of the compounds were performed on structures where the cell parameters were strained by +/-0.02%.

### 4.3. Semiempirical Model of Thermal Conductivity

The same form of the semiempirical model used by Miller et al. [37] was used in this study (Figure 5), but the exponents were reoptimized (using a gradient decent algorithm) since we use different definitions of $v_{\text{s}}$ and γ. Explicitly, the experimental lattice thermal conductivity $\kappa_{\text{L}}$ at 300 K is estimated as

(15)κL=2.69×10−3m¯vs4.169γVa0.498N0.935+32π61/3kBvsVa0.6951−1N2/3,

where $\bar{m}$ is the average atomic mass, $v_{\text{s}}$ is the RMS speed of sound, γ is the harmonic Grüneisen parameter found from the speed of sound ratio (equation (8)), $V_{\text{a}}$ is the average atomic volume, and N is the number of atoms per primitive unit cell. All quantities were found from the Materials Project [55] database and need to be in SI units for equation (15). The longitudinal and transverse speeds of sound were obtained from the theoretical density ρ and Voigt-Reuss-Hill values [56] of bulk modulus $B_{\text{VRH}}$ and shear modulus $G_{\text{VRH}}$ through the relations [34]:

(16)vt=GVRHρ,(17)vl=BVRH+4/3GVRHρ.

Since only structural and harmonic material descriptors are used, equation (15) can be referred to as a “harmonic thermal conductivity.” A similar equation was also found to reasonably estimate the 300 K values of lattice thermal conductivity calculated by DFT (Supplemental Note 5).

## Data Availability

Any relevant data not contained within this publication is available from the corresponding authors upon reasonable request.

## Additional Points

*Broader Context*. Nonlinear “anharmonic” forces between atoms are responsible for many behaviors of thermoelectric, battery, fuel cell, and photovoltaic materials, because they govern diverse phenomena ranging from the thermodynamics of heat to the propagation of earthquakes. Yet, the ubiquity of anharmonicity does not mean that it is well understood. Without a detailed study of the vibrational properties of a material (i.e., phonons), it is nearly impossible to make any prediction about the magnitude of thermal expansion, thermal conductivity, or other so-called anharmonic properties of solids. Here, we demonstrate a fundamental relation between harmonicity (e.g., elasticity), which is well characterized in every kind of solid, and anharmonicity, which is not well characterized and is a highly active field of research. We show how anharmonicity can be interpreted as the result of mechanical pressure exerted by vibrating atoms on the rest of the solid and contextualize this perspective with the current quantum mechanical interpretation. This work provides a new framework by which anharmonicity may be characterized from harmonic properties of solids, subsequently leading to metrics of anharmonicity useful for material screening and design.

## Supplementary Materials

Supplementary Materials Supplemental Note 1: momentum flux derivation of pressure. Supplemental Note 2: thermodynamic pressure. Supplemental Note 3: formulation of the Grüneisen parameter in terms of the speeds of sound. Supplemental Note 4: average speed of sound ratio and density. Supplemental Note 5: estimation of thermal conductivity from the harmonic Grüneisen parameter. Figure S1: estimation of lattice thermal conductivity using the harmonic Grüneisen parameter. Figure S2: residual plots for material descriptors used in the semiempirical model. Figure S3: residual plots for material descriptors used in the semiempirical model.
