# Supplementary material for: Inherent Anharmonicity of Harmonic Solids
Source: Research (Wash D C). 2022 Apr 29;2022:9786705. doi: 10.34133/2022/9786705 (PMC11014735; doi:10.34133/2022/9786705)
Supplement: Supplementary Materials — Supplemental Note 1: momentum flux derivation of pressure. Supplemental Note 2: thermodynamic pressure. Supplemental Note 3: formulation of the Grüneisen parameter in terms of the speeds of sound. Supplemental Note 4: average speed of sound ratio and density. Supplemental Note 5: estimation of thermal conductivity from the harmonic Grüneisen parameter. Figure S1: estimation of lattice thermal conductivity using the harmonic Grüneisen parameter. Figure S2: residual plots for material descriptors used in the semiempirical model. Figure S3: residual plots for material descriptors used in the semiempirical model. [file 9786705.f1.pdf]

Supplemental Information

Agne, M.T. et al. Inherent anharmonicity of harmonic solids

Supplemental Note 1

Momentum flux derivation of pressure

The pressure P exerted on an imaginary surface in the interior of a gas is identical to the pressure the gas exerts on the walls of the container. This pressure due to kinetic particles having mass m and velocity v can be calculated by considering their flux of linear momentum through the arbitrary surface. For simplicity, consider the one-dimensional case where particles only move left and right. Then, the particle flux going through the surface to the right is $j_\mathrm{R} = n_\mathrm{R} v_\mathrm{R}$ (particles/second/area) where n_R is the number density of particles moving to the right (particles/volume) with velocity v_R. These particles have a momentum (also moving to the right) $p_\mathrm{R} = m v_\mathrm{R}$, so that the flux of momentum (to the right) is $J_\mathrm{R} = j_\mathrm{R} p_\mathrm{R}$. In the same way, the flux of momentum moving through the plane to the left is $J_\mathrm{L} = j_\mathrm{L} p_\mathrm{L}$. At steady state there will be an equal number of particles moving left and right, such that $n_\mathrm{R} = n_\mathrm{L} = n/2$ where n is the total number density of particles. Also at steady state $|v_\mathrm{L}| = |v_\mathrm{R}| = |v|$, so that the thermodynamic (time-averaged) pressure can be calculated as the sum of the constituent momentum fluxes as [1,2]

$$P = J_\mathrm{R} + J_\mathrm{L} = \frac{n}{2} v_\mathrm{R}(m v_\mathrm{R}) + \frac{n}{2} v_\mathrm{L}(m v_\mathrm{L}) = \rho |v|^2,$$

where ρ is the bulk mass density of the particles. Of course, $\rho |v|^2$ is related to the average kinetic energy density as

$$\frac{KE}{V} = \frac{\rho |v|^2}{2}.$$

In the case of particles moving in arbitrary directions relative to the surface, the methodology is the same but the velocity is scaled by the cosine of the angle between the incident direction of the particle and the normal direction of the surface.

Since the kinetic energy of vibrating atoms in a solid ($mv^2/2$) is due to the contributions of the excited phonon modes whose kinetic energy is related to the frequency of the vibration ($\hbar\omega/2$), this provides the connection to the phonon pressure model described in the Methods of the main text.

Supplemental Note 2

Thermodynamic pressure

The study of thermal expansion is, inherently, the study of how the interface of a material (relative to its surroundings) moves with temperature. This is what a dilatometry measurement does. Thermodynamically, this can be described by a two-phase system (α and β) having an interface (Σ). Here, we can think of α as the solid, β as the surroundings (e.g. a vacuum), and Σ as the surface of the solid. We consider a case where the exchange of atoms between α and β is negligible, such that the position of the interface does not depend on mass transport. Thermal expansion is thus the change in the equilibrium position of the interface as a function of temperature.

We start by defining the total energy and total entropy of the two-phase system as the sum of the per volume quantities (E_v and S_v) for α and β, multiplied by their respective volumes (V), and the per area quantities of Σ, multiplied by the total area (A), as [3,4]

$$E = E_v^{\alpha} V^{\alpha} + E_v^{\beta} V^{\beta} + \mathrm{E}_A^{\sigma} A \qquad \text{(S1)}$$

$$S = S_v^{\alpha} V^{\alpha} + S_v^{\beta} V^{\beta} + \mathrm{S}_A^{\sigma} A \, . \qquad \text{(S2)}$$

To find the conditions that give rise to thermodynamic equilibrium we use the internal energy, which is an energy function that does not make any assumptions about the conditions of the intensive variables at equilibrium. The equilibrium condition requires that the first variation of the internal energy has to be zero ($\delta E = 0$), with the constraints that the total entropy and total volume are constant.

To find the equilibrium condition under these constrains, we use the method of Lagrange Multipliers commonplace in thermodynamics to define the energy function:

$$E^{*} = E - \lambda_T (S - C_S) \qquad \text{(S3)}$$

that uses the Lagrange multiplier λ_T and C_S is the total entropy at equilibrium (a constant such that $S - C_S = 0$). The variation of this energy is

$$\delta E^* = \delta E - \lambda_T \delta S \qquad \text{(S4)}$$

where

$$\delta E = V^\alpha \delta E_v^\alpha + E_v^\alpha \delta V^\alpha + V^\beta \delta E_v^\beta + E_v^\beta \delta V^\beta + E_A^\Sigma \delta A + A \delta E_A^\Sigma \qquad \text{(S5)}$$

and

$$\delta S = V^\alpha \delta S_v^\alpha + S_v^\alpha \delta V^\alpha + V^\beta \delta S_v^\beta + S_v^\beta \delta V^\beta + S_A^\Sigma \delta A + A \delta S_A^\Sigma\,. \qquad \text{(S6)}$$

Substituting Eqs. S5 and S6 into Eq. S4 and rearranging,

$$\delta E^* = V^\alpha(\delta E_v^\alpha - \lambda_T \delta S_v^\alpha) + V^\beta\left(\delta E_v^\beta - \lambda_T \delta S_v^\beta\right) + A\left(\delta E_A^\Sigma - \lambda_T \delta S_A^\Sigma\right) + (E_v^\alpha - \lambda_T S_v^\alpha)\,\delta V^\alpha + (E_v^\beta - \lambda_T S_v^\beta)\delta V^\beta + (E_A^\Sigma - \lambda_T S_A^\Sigma)\delta A\,, \qquad \text{(S7)}$$

which is equal to zero at equilibrium. At constant volume it is well-known that $\delta E = T\delta S$, and so all the terms at constant volume (and area) are zero when $\lambda_T = T$, which is to say that one equilibrium condition is that the system be at constant temperature. The other equilibrium condition is found from the variations of the volumes and interfacial area as

$$\delta E^* = (E_v^\alpha - TS_v^\alpha)\delta V^\alpha + \left(E_v^\beta - TS_v^\beta\right)\delta V^\beta + \left(E_A^\Sigma - TS_A^\Sigma\right)\delta A = 0\,, \qquad \text{(S8)}$$

where the quantity in brackets is the per volume Grand Canonical free energy [5] (or Grand Potential), $\Omega_v = -P$, which is the pressure of each phase at temperature T. The per area quantity is an interfacial energy that is the excess Grand canonical free energy per area. Then we can write Eq. S8 as

$$\delta E^* = \Omega_v^\alpha \delta V^\alpha + \Omega_v^\beta \delta V^\beta + \Omega_A^\Sigma \delta A = 0, \qquad \text{(S9)}$$

and considering that the volumes and area are not independent variables, but are geometrically coupled (i.e. $\delta V^\beta = -\delta V^\alpha$ and $\delta A = cA^{-1/2}\,\delta V^\alpha$, where $cA^{-1/2} = (\partial A/\partial V^\alpha)$ depends on the exact geometry but c is a constant for simple geometries like cubes and spheres), then

$$\left(\Omega_v^\alpha - \Omega_v^\beta + cA^{-1/2}\Omega_A^\Sigma\right)\delta V^\alpha = 0 \qquad \text{(S10)}$$

which means that the equilibrium condition is

$$\Omega_v^\alpha - \Omega_v^\beta + cA^{-\frac{1}{2}}\Omega_A^\Sigma = 0\,. \qquad \text{(S11)}$$

When the second phase, i.e. the surroundings, does not exert a significant pressure (such as a vacuum), then $\Omega_v^\beta \approx 0$ and Eq. S11 can be written as Eq. 4 in the main text. This is to say that the elastic pressure is really a characterization of the interfacial energy (i.e. $P_{elasic} = -cA^{-\frac{1}{2}}\Omega_A^\Sigma$). Note that main text Eq. 4 made use of the relation

$$P_{elastic} = -B\frac{V-V_0}{V} = V\frac{\partial}{\partial V}\left[\left(\frac{\partial E}{\partial A}\right)\left(\frac{\partial A}{\partial V}\right)\right]\frac{V-V_0}{V}\,, \qquad \text{(S12)}$$

which allows for the interfacial quantities to be written using the notation of a bulk modulus $B = -V\frac{\partial}{\partial V}\left(\frac{\partial E}{\partial V}\right)$. This relation holds when $\frac{\partial}{\partial V}\left[\left(\frac{\partial E}{\partial A}\right)\left(\frac{\partial A}{\partial V}\right)\right] \approx \frac{\mathrm{d}P}{\mathrm{d}V} = \frac{P_{elastic} - 0}{V - V_0}$, where V_0 is a reference volume where the elastic pressure is zero.

It is important to note that the Gibbs free energy function is not appropriate for this analysis because it presupposes that equilibrium is achieved when both α and β are at the same pressure (i.e. $\Omega_v^\alpha = \Omega_v^\beta$), which is not the same as the balance of pressures derived here (Eq. S11). Similarly, the Helmholtz free energy function is for determining equilibrium conditions when the volume is held constant, which is not the case for thermal expansion.

Supplemental Note 3

Formulation of the Gruneisen parameter in terms of the speeds of sound

The scalar estimate for the Gruneisen parameter, derived using the analytical model in Methods,

$$\gamma \approx \frac{3}{2}\frac{B}{\rho v_s^2}, \tag{S13}$$

can be written in terms of the ratio ($x = v_\mathrm{t}/v_\mathrm{l}$) of transverse and longitudinal speeds of sound, respectively. Making use of the definition of the shear modulus $G = \rho v_\mathrm{t}^2$, Eq. S13 can be rewritten as

$$\gamma \approx \frac{3}{2}\frac{B}{G}\left(\frac{\rho v_\mathrm{t}^2}{\rho v_s^2}\right), \tag{S14}$$

and since the ratio $\frac{B}{G} = \frac{2}{3}\frac{(1+\nu)}{(1-2\nu)}$ can be written as solely a function of the Poisson ratio ν, which is defined by the ratio x as [6]

$$\nu = \frac{1}{2}\frac{\left[\left(\frac{1}{x}\right)^2 - 2\right]}{\left[\left(\frac{1}{x}\right)^2 - 1\right]}. \tag{S15}$$

Then, considering the average speed of sound as the RMS sound velocity,

$$v_s^2 = \frac{1}{3}(v_\mathrm{l}^2 + 2v_\mathrm{t}^2), \tag{S16}$$

it is then possible to get the simple form:

$$\gamma \approx \frac{3}{2}\frac{(3-4x^2)}{(1+2x^2)}, \tag{S17}$$

which has a root ($\gamma = 0$) at $x = \frac{\sqrt{3}}{2}$, corresponding to the upper thermodynamic limit of stability for the ratio G/B for a homogeneous isotropic linear elastic material. Clearly, $x = 0$ results when $v_\mathrm{t} \to 0$, which is often considered as a property of a liquid.

Using the definitions of $B = \rho\left(v_l^2 - \frac{4v_t^2}{3}\right)$ and $v_\mathrm{s} = \left(\frac{1}{3}\left(\frac{2}{v_t^3} + \frac{1}{v_l^3}\right)\right)^{-1/3}$ as described by Anderson[7] gives an estimate of the Gruneisen parameter with a functional dependence on x as:

$$\gamma = \frac{3}{2}\frac{\left(1-\frac{4}{3}x^2\right)}{\left(\frac{1}{3}\left(\frac{2}{x^3}+1\right)\right)^{-2/3}}. \qquad \text{(S18)}$$

This result diverges rapidly as $x \to 0$ because the average speed of sound, v_s, goes to zero as v_t goes to zero. This is unphysical in the sense that there is an average speed of sound in liquids even though $v_\mathrm{t} = 0$. Thus, the root mean square speed of sound is used as a more physical representation of the average speed of sound in the limit as solids lose their capacity to sustain shear elastic waves.

Connection with the Cauchy condition

Eq. S17 can also be written in terms of the C_{11}, C_{12} and C_{44} elastic constants for a material with cubic symmetry (including homogeneous polycrystals where random grain orientation effectively averages any material to have cubic symmetry), as [8]

$$\gamma \approx \frac{3}{2}\frac{(C_{11}+2C_{12})}{(C_{11}+2C_{44})}, \qquad \text{(S19)}$$

which suggests that $\gamma \approx 1.5$ for a material that obeys the Cauchy condition $C_{12} = C_{44}$. The idea of Cauchy pressure ($P_C = C_{12} - C_{44}$) as a metric for bonding anisotropy leads us to rearrange Eq. S19 as

$$\gamma \approx \frac{3}{2} + \frac{3P_C}{C_{11}+2C_{44}}. \qquad \text{(S20)}$$

It is bond anisotropy, after all, that gives rise to the ratio x of the bulk speeds of sound. In this terminology, $P_C = 0$ is indicative of ionic bonding (spherical pair-wise interactions), whereas $P_C > 0$ is typical of more metallic (delocalized) bonding and $P_C < 0$ is expected in covalent (directional) bonds [9].

Gruneisen parameter in 1D

Throughout this work we have been discussing 3D solids, where atoms vibrate in 3 dimensions. This gives rise to the coefficient (3/2) in main text Eq. 6, where the 3 comes from $\alpha = 3\alpha_{\mathrm{L}}$ (relating the volumetric and linear thermal expansion coefficients for cubic symmetry) and the factor of ½ comes from the average incidence cosine. In 1D, however, there is only the linear thermal expansion coefficient α_{L} and the atoms all vibrate in the same direction so that $|\hat{e} \cdot \hat{n}| = 1$. Including these considerations in the phonon pressure model allows for an estimation of the Gruneisen parameter in 1D.

Considering the generalized thermodynamic definition of the Gruneisen parameter tensor [12], the 1D Gruneisen parameter is

$$\gamma_{\mathrm{1D}} = L\left(\frac{\partial F}{\partial E}\right)_L = \frac{\alpha_{\mathrm{L}} B_{\mathrm{1D}}}{c_{\mathrm{L}}}$$

where $\alpha_L = d\ln(L)/dT$ (at constant force F), $B_{\mathrm{1D}} = -L\,(\partial F/\partial L)_T$ can be thought of as the isothermal 1D "bulk modulus" and c_{L} is the (per length) heat capacity at constant L (length).

The phonon pressure model can be used to estimate α_{L} using the same force-balance equation as main text Eq. 4 and Eq. 9, considering an incidence cosine of 1 and that all quantities per volume in 3D are per length in 1D. This leads to

$$\alpha_{\mathrm{L}} = \frac{c_{\mathrm{L}}}{\rho_{\mathrm{L}} v_{\mathrm{1D}}^2}$$

and since there is only 1 speed of sound v_{1D} (in the 1D Debye approximation), which defines the 1D bulk modulus as $B_{\mathrm{1D}} = \rho_{\mathrm{L}} v_{\mathrm{1D}}^2$, where ρ_{L} is the linear mass density, then we conclude that $\gamma_{\mathrm{1D}} = 1$. This suggests that even a harmonic linear chain of atoms has a thermal expansion, as well as a Gruneisen parameter that is apparently material independent. It is interesting to note that $\gamma_{\mathrm{1D}} = 1$ is also obtained considering a linear chain with pairwise interactions described by the Morse potential, $V(A) \propto [1 - \exp(-a(A - A_0))]^2$, when the scaling parameter $a = 2/(3A_0)$, where A_0 is the reference equilibrium interatomic distance (see Eq. 16 of Dugdale and MacDonald[13]). Future work to understand the connections between bond strength and anisotropy in the magnitude of the Gruneisen parameter may benefit by considering the effects at different dimensions.

Supplemental Note 4

Average speed of sound ratio and density

In Table S1, the values used to determine the yellow-green circles plotted in Fig. 4b can be found.

Table S1. Compounds binned according to their speed of sound ratio $x = v_\mathrm{t}/v_\mathrm{l}$, having average Gruneisen parameter γ and density ρ.

# of compounds	Average $v_\mathrm{t}/v_\mathrm{l}$	Average γ	Average ρ (kg m^{-3})
2	0.275	3.0	13709
4	0.364	2.5	9224
20	0.467	1.9	6636
101	0.563	1.6	6073
36	0.627	1.0	4124

Supplemental Note 5

Estimation of thermal conductivity from the harmonic Gruneisen parameter

The estimation of the thermodynamic Gruneisen parameter from harmonic phonon calculations (i.e. second order interatomic force constants) provides a basis for computationally efficient estimations of anharmonic material properties, such as thermal expansion and thermal conductivity. Here, we use a semi-empirical thermal conductivity estimation to predict the lattice thermal conductivity at 300 K obtained by Density Functional Theory calculations for 119 compounds [10]. Using a reoptimized form of the equation presented by Miller, et al. [11], we find that the semi-empirical equation

$$\kappa_L = 2.69 \times 10^{-3} \frac{\bar{m} v_s^{4.142}}{\gamma\, V^{0.524}\, N^{1.136}} + \left(\frac{3}{2}\right)\left(\frac{\pi}{6}\right)^{1/3} \frac{k_{\mathrm{B}} v}{V^{0.689}}\left(1 - \frac{1}{N^{2/3}}\right) \quad \text{(S21)}$$

can be used, where $\bar{m}$ is the average atomic mass, v_s is the RMS speed of sound, γ is the harmonic Gruneisen parameter found from the speed of sound ratio, V_a is the average atomic volume, and N is the number of atoms per primitive unit cell. The longitudinal and transverse speeds of sound were estimated from DFT calculated bulk and shear moduli and the density according to Anderson [7]. This simple estimation accurately predicts the DFT calculated lattice thermal conductivity of ~83% of the compounds within a factor of 3, most of which are within a factor of 2, thus providing the basis for high-throughput estimations of DFT calculated lattice thermal conductivity from only speed of sound (Figure S1). Further improvement of the accuracy of the semi-empirical model may be possible, but there is not a clear indication which material descriptor should be targeted (Figure S2 and S3). Nevertheless, the similar values of the exponents in main text Eq. 15 and those in Eq. S21 indicates the robust ability of the semi-empirical model to estimate both experimental and DFT calculated lattice thermal conductivity values using a consistent set of descriptors. Here, we use a harmonic estimate for the Gruneisen parameter that leads to a harmonic estimate of thermal conductivity. The accessibility of speed of sound values, both experimentally and computationally, makes this estimate of thermal conductivity a first step towards accurate, high-throughput estimations of lattice thermal conductivity using the harmonic Gruneisen parameter, however more complex estimations (including machine learning) may be implemented [10].

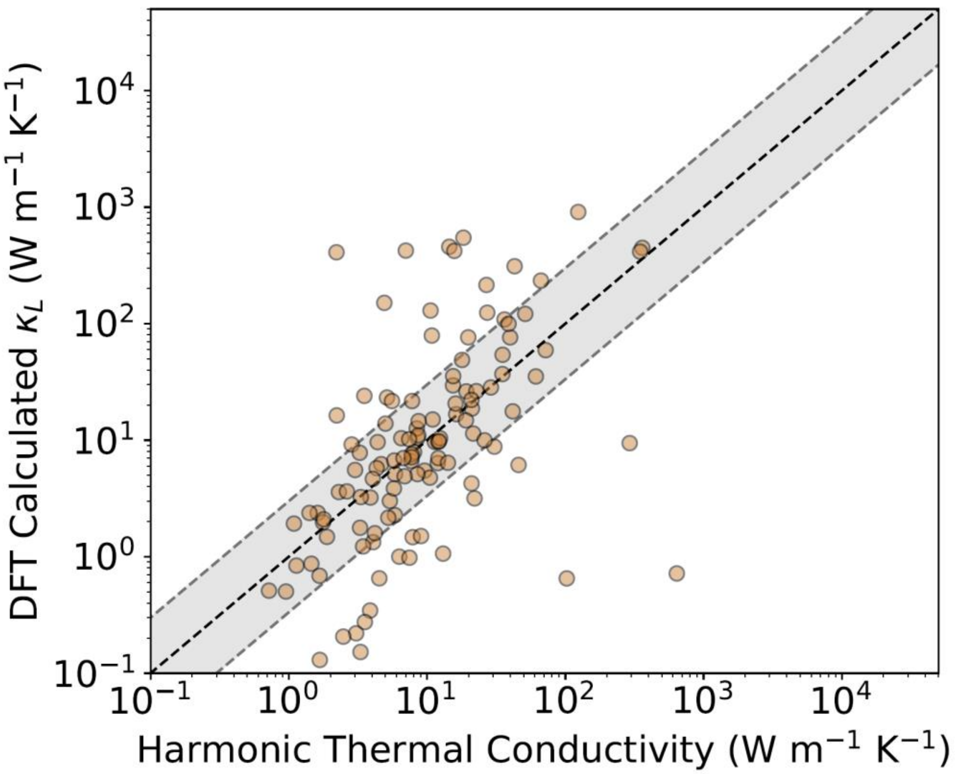

Figure S1. Estimation of lattice thermal conductivity using the harmonic Gruneisen parameter. An equals plot showing the predictive power of the semi-empirical model to estimate the DFT calculated 300 K lattice thermal conductivity of 119 previously reported compounds [10]. The light gray dashed lines indicate a factor of 3 from the equal line. Over 80% of the compounds are accurate within a factor of 2.

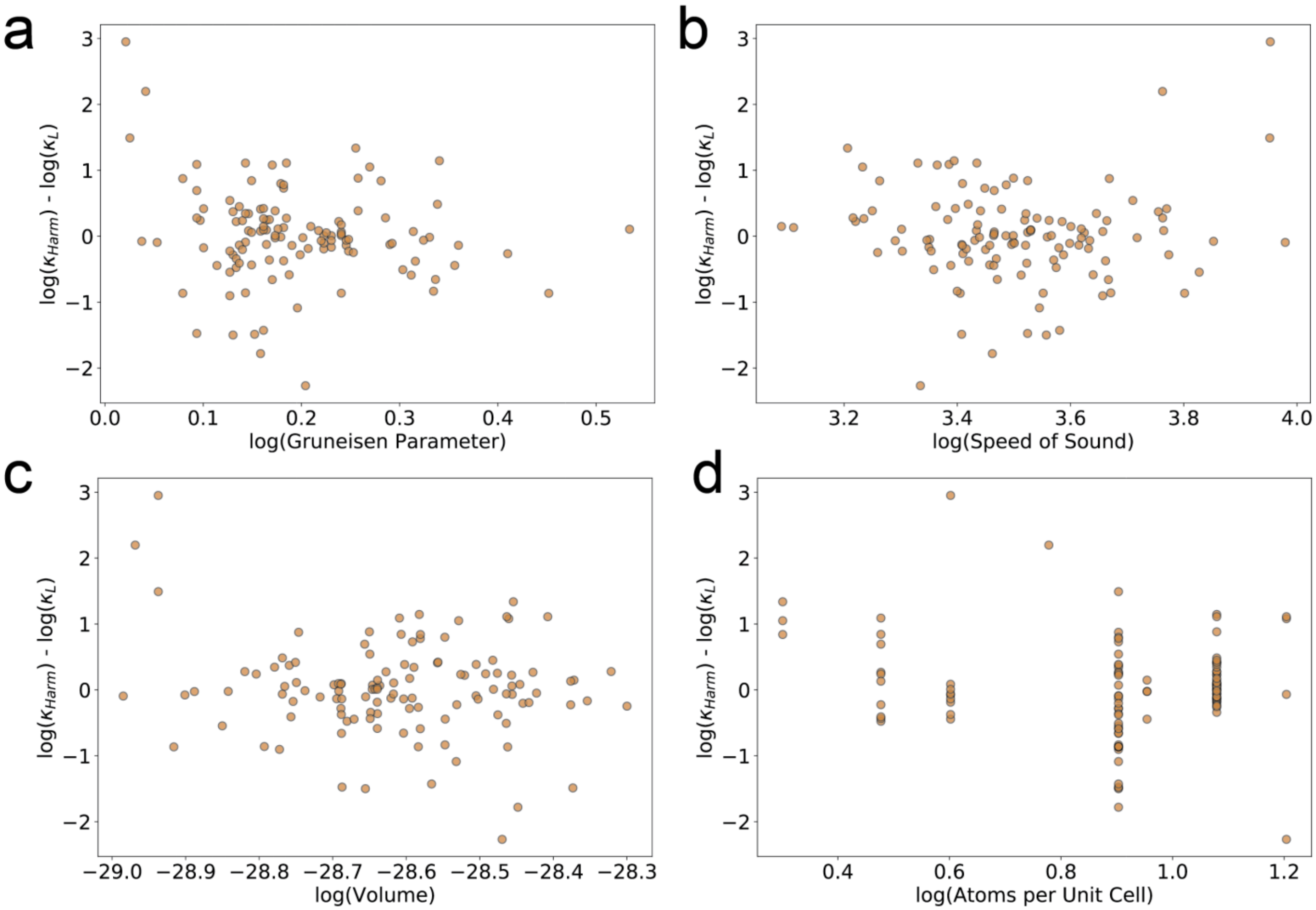

Figure S2. Residuals plots for material descriptors used in the semi-empirical model. There is no clear trend in the residuals for the 300 K DFT thermal conductivity prediction as a function of (a) the harmonic Gruneisen parameter, (b) the RMS speed of sound, (c) the unit cell volume, or (d) the number of atoms per unit cell.

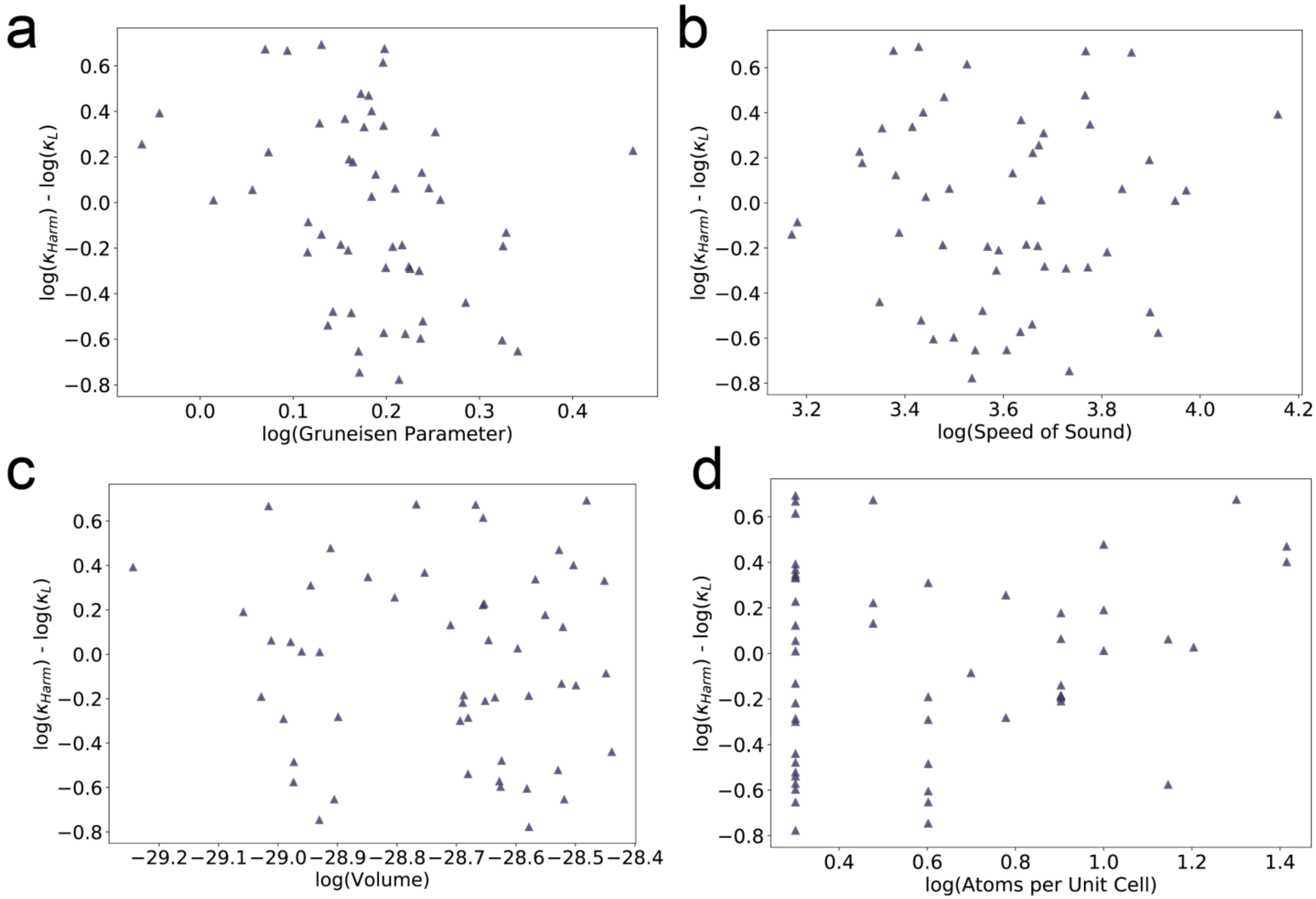

Figure S3. Residuals plots for material descriptors used in the semi-empirical model. There is no clear trend in the residuals for the 300 K experimental lattice thermal conductivity prediction (main text Fig. 5) as a function of (a) the harmonic Gruneisen parameter, (b) the RMS speed of sound, (c) the unit cell volume, or (d) the number of atoms per unit cell.

References

1. DiSessa, A.A., Momentum flow as an alternative perspective in elementary mechanics. American Journal of Physics, 1980. 48(5): p. 365-369.
2. Herrmann, F. and G.B. Schmid, Statics in the momentum current picture. American Journal of Physics, 1984. 52(2): p. 146-152.
3. Bokstein, B.S., M.I. Mendelev, and D.J. Srolovitz, Thermodynamics and kinetics in materials science: a short course. 2005: Oxford University Press.
4. Chen, L.-Q., Phase-field models for microstructure evolution. Annual review of materials research, 2002. 32(1): p. 113-140.
5. Landau, L. and E. Lifshitz, Statistical Physics, part 1. 1980, Pergamon Press.
6. Greaves, G.N., et al., Poisson's ratio and modern materials. Nature Materials, 2011. 10(11): p. 823-837.
7. Anderson, O.L., A simplified method for calculating the debye temperature from elastic constants. Journal of Physics and Chemistry of Solids, 1963. 24(7): p. 909-917.
8. Leont'ev, K., O svyazi uprugikh i teplovykh svoistv veshchestv [Bonding of elastic and thermal properties of substances]. Akusticheskii zhurnal-Acoustical Physics, 1981: p. 554-561.
9. Eberhart, M.E. and T.E. Jones, Cauchy pressure and the generalized bonding model for nonmagnetic bcc transition metals. Physical Review B, 2012. 86(13): p. 134106.
10. Juneja, R., et al., Coupling the High-Throughput Property Map to Machine Learning for Predicting Lattice Thermal Conductivity. Chemistry of Materials, 2019. 31(14): p. 5145-5151.
11. Miller, S.A., et al., Capturing Anharmonicity in a Lattice Thermal Conductivity Model for High-Throughput Predictions. Chemistry of Materials, 2017. 29(6): p. 2494-2501.
12. Key, S. W. Grüneisen tensor for anisotropic materials. J. Appl. Phys. 38, 2923–2928 (1967).
13. Dugdale, J. S. & MacDonald, D. K. C. Vibrational anharmonicity and lattice thermal properties. Phys. Rev. 96, 57–62 (1954).
